# PAX6/CXCL14 regulatory axis promotes the repair of corneal injury by enhancing corneal epithelial cell proliferation

**DOI:** 10.1186/s12967-024-05270-z

**Published:** 2024-05-15

**Authors:** Ruijue Ma, Yingxi Li, Xiaoli Dong, Yiming Zhang, Xiaosu Chen, Yue Zhang, Haohan Zou, Yan Wang

**Affiliations:** 1 Tianjin Eye Institute, Tianjin Key Lab of Ophthalmology and Visual Science, Tianjin Eye Hospital, Nankai University Affiliated Eye Hospital, Tianjin, 300020 China; 2 Department of Pathogen Biology, School of Basic Medical Sciences, Tianjin Medical University, Tianjin, 300070 China; 3 Translational Health Sciences, Bristol Medical School, University of Bristol, Bristol, BS8 1UD UK; 4 Clinical College of Ophthalmology, Tianjin Medical University, Tianjin, 300070 China; 5 School of Medicine, Nankai University, Tianjin, 300071 China

## Abstract

**Background:**

Corneal injuries, often leading to severe vision loss or blindness, have traditionally been treated with the belief that limbal stem cells (LSCs) are essential for repair and homeostasis, while central corneal epithelial cells (CCECs) were thought incapable of such repair. However, our research reveals that CCECs can fully heal and maintain the homeostasis of injured corneas in rats, even without LSCs. We discovered that CXCL14, under PAX6’s influence, significantly boosts the stemness, proliferation, and migration of CCECs, facilitating corneal wound healing and homeostasis. This finding introduces CXCL14 as a promising new drug target for corneal injury treatment.

**Methods:**

To investigate the PAX6/CXCL14 regulatory axis’s role in CCECs wound healing, we cultured human corneal epithelial cell lines with either increased or decreased expression of PAX6 and CXCL14 using adenovirus transfection in vitro. Techniques such as coimmunoprecipitation, chromatin immunoprecipitation, immunofluorescence staining, western blot, real-time PCR, cell colony formation, and cell cycle analysis were employed to validate the axis’s function. In vivo, a rat corneal epithelial injury model was developed to further confirm the PAX6/CXCL14 axis’s mechanism in repairing corneal damage and maintaining corneal homeostasis, as well as to assess the potential of CXCL14 protein as a therapeutic agent for corneal injuries.

**Results:**

Our study reveals that CCECs naturally express high levels of CXCL14, which is significantly upregulated by PAX6 following corneal damage. We identified SDC1 as CXCL14’s receptor, whose engagement activates the NF-κB pathway to stimulate corneal repair by enhancing the stemness, proliferative, and migratory capacities of CCECs. Moreover, our research underscores CXCL14’s therapeutic promise for corneal injuries, showing that recombinant CXCL14 effectively accelerates corneal healing in rat models.

**Conclusion:**

CCECs play a critical and independent role in the repair of corneal injuries and the maintenance of corneal homeostasis, distinct from that of LSCs. The PAX6/CXCL14 regulatory axis is pivotal in this process. Additionally, our research demonstrates that the important function of CXCL14 in corneal repair endows it with the potential to be developed into a novel therapeutic agent for treating corneal injuries.

**Supplementary Information:**

The online version contains supplementary material available at 10.1186/s12967-024-05270-z.

## Introduction

The cornea, serving as a vital component of both the ocular surface barrier and refractive system, is susceptible to various forms of trauma and pathological damage [1–3]. This vulnerability leads to a high incidence of corneal injuries, affecting approximately 20% of the global population at least once in their lifetime. Inadequate corneal repair results in corneal opacity, keratinization, neovascularization, and ultimately, corneal blindness [5]. Corneal repair is a complex process involving multiple corneal tissue cells. Its complete regulatory mechanism has not been fully elucidated [6]. Maintaining the physiological dynamic balance between corneal epithelial cell proliferation and replacement is particularly crucial for preserving corneal transparency [7].

The homeostasis of the corneal epithelium is well explained by Thoft and Friend’s classical *XYZ* theory: the sum of corneal epithelial basal cells (*X*) and peripheral centripetal motor cells (*Y*) equals the cells lost on the corneal surface (*Z*) [8]. The mechanism of corneal epithelial repair has primarily focused on the significant role of limbal stem cells (LSCs, *Y*) in this process [9, 10]. Repair has focused on limbal stem cells (LSCs) in the Vogt palisades [11, 12]. Corneal injury was thought to stimulate the proliferation of LSCs, promoting corneal repair [13]. Nevertheless, the absence of clear, positive markers for LSCs means their presence is only indirectly evidenced, casting doubt on their essential role in corneal repair—a matter that continues to spark debate [14].

Recent studies have challenged the exclusive role of LSCs in corneal repair. For example, the central cornea can undergo immediate and significant repair after injury [16], and studies have reported successful corneal healing and maintenance of clarity without LSC contribution [17, 19]. Such researches points toward the hitherto underappreciated significance of CCECs in the response to corneal injuries. Moreover, Huang et al. demonstrated that despite creating a limbal epithelial defect model in rabbits, corneal clarity was restored in the majority of cases [17]. Chang et al. reported complete corneal injury repair and limbal coverage by corneal epithelial cells (CCECs) following LSC ablation using an excimer laser [18]. Majo et al. created a limbal epithelial defect model in mice, finding that the cornea maintain transparency for at least four months [19]. Additionally, Kwkita et al. transplanted stainless steel rings around the cornea of rabbits to impede communication and migration between LSCs and CCECs, revealing that the central cornea remained normal for at least six months [15]. Meanwhile, some studies have found that it generally takes 4 to 5 months for LSCs to proliferate and migrate to the center of the cornea, but central corneal can be repaired immediately and significantly after injury [15]. These findings affirm the importance of central corneal epithelial cells (CCECs) in corneal repair and their capability to maintain post-injury corneal stability. Yet, the precise mechanisms of CCECs’ functions are not fully understood. A deeper investigation into the reasons CCECs can act independently in corneal repair is urged, potentially yielding new insights for therapeutic strategies in corneal injury recovery.

In this study, we’ve demonstrated for the first time that CCECs exert their independent corneal repair function through the PAX6/CXCL14 regulatory axis. Following corneal injury, the transcriptional activation of CXCL14 by PAX6 promotes the function of CCECs. Our findings demonstrate the significant roles of CXCL14 in enhancing stemness, proliferation, and migration abilities of CCECs. CXCL14 is an inherently highly expressed secretory protein in the cornea, exerting a direct effect on corneal tissue. Compared to existing treatments for corneal injuries, this autologous protein is likely to have fewer side effects, making CXCL14 a promising therapeutic target for patients with corneal injuries. With the aim of achieving high efficiency and low side effects, green nanomaterials have become a research focus as carriers for eye drops [49, 50]. The potential future integration of CXCL14 with nanomaterials could play a significant role in the treatment of corneal injuries.

## Materials and methods

### Rat models and related experimental operations

Healthy male SD rats weighing approximately 250 g were used for the experiment. All animal studies were approved by the Ethics Committee of the Tianjin Eye Hospital and were conducted by skilled experimenters according to an approved protocol in accordance with the principles and procedures outlined in the NIH Guide for the Care and Use of Laboratory Animals. Under the microscope, corneal epithelium was mechanically scraped with corneal epithelial scrapers used for LASEK. CXCL14 antibody (Abcam, Cambridge, UK) and PAX6 antibody (Abcam, USA) were used for immunohistochemistry staining.

To verify whether the central corneal epithelium (CCE) has a repairing effect during corneal injury, we established 3 corneal injury models by mechanical curettage (with 8 rats in each model group): the CCE curettage group, the LE curettage group, and the whole corneal epithelium (WCE) curettage group. The calculation formula of corneal healing rateis: healing rate = repair area/damage area × 100%. Haze was assessed according to the evaluation method of the corneal injury experiment by Yoeruek et al. [20]. Corneal haze was divided into 5 grades: 0, completely transparent; 1, extremely mild haze, with the iris and pupil clearly visible; 2, mild haze, with the iris and pupil still visible; 3, moderate haze, with the iris and pupil faintly visible; and 4, severe haze, with the iris and pupil not clearly seen. Neovascularization was assessed by using a slit lamp and statistical results were obtained by analyzing tissue sections under a microscope (20 ×). These characteristics were observed at 0 h, 12 h, 24 h, 48 h, 72 h, 96 h, 1 w, 1 m, 3 m, and 6 m after operation under the slit lamp with normal light or cobalt blue light after fluorescein sodium staining.

To investigate which genes promote the repair of corneal injury in CCE, we established a model of LE scrape injury in rats (3 cases). After 24 h, CCE of the operative eye and the contralateral normal eye in the same rat were taken, and the genomes of these tissues were sequenced and analyzed.

To verify the expression of *CXCL14* in the cornea, the corneal and kidney tissues of the same normal rat were evaluated by immunohistochemistry (4 cases), real-time polymerase chain reaction (PCR) (8 cases), and Western blot analysis (8 cases).

We also constructed stable corneal models with CXCL14 overexpression or downregulation by using lentivirus eye drops in rats, and the results of immunohistochemistry showed that the model was constructed successfully and was stable (Supplementary Fig. 1). Then, we scraped the LE in each model, we observed the healing and corneal state under the slit lamp at 0 h, 12 h, 24 h, 48 h, 72 h, 96 h, 1 w, 1 m, etc., after operation, under the conditions of common illumination and cobalt blue light after fluorescein sodium staining (Fig. 2F–H).

### Cell culture related experimental operations

HCE-2 (50, B1) cells and rat primary corneal epithelial cells were cultured in Dulbecco’s Modified Eagle’s Medium (Nutrient Mixture F-12 (DMEM/F12)), supplemented with 10% fetal bovine serum, 100 U/mL penicillin, and 100 mg/mL streptomycin (Gibco, Grand Island, NY, USA) at 37 °C and 5% CO_2_. 293 T cells were cultured in DMEM supplemented with 10% FBS, 100 U/mL penicillin, and 100 mg/mL streptomycin.

We construct stable cell lines with *CXCL14* overexpression or downregulation in human corneal epithelial cells (HCECs) (HCE-2 [50, B1]), with corresponding control cells, by the method of stable transfection with lentiviral packaging, and we verified the success of the cell line construction by Western blot analysis (Fig. 2I). Then, we used the stable HCEC lines to carry out the wound healing assay. We took photos of the cells at the time points of 0 h, 24 h, and 48 h under the microscope, and we measured the healing area.

To explore the mechanism by which *CXCL14* promotes corneal repair after injury, we used HCE- 2 (50, B1) cells with *CXCL14* overexpression or downregulation to perform soft agar assay. We inoculated 6-well plates with 800 cells per well, with 3 holes for each group. We fixed and stained the cells with crystal violet after 7 days of culture, counted the cells, and performed statistical analysis.

To confirm whether CXCL14 mediates corneal epithelial cell repair after injury through NF-κB signaling, we stimulated HCE-2 with recombinant CXCL14 (20 ng/mL) for 4 h. After co-culture for 0 h, 0.5 h, 1 h, 2 h, and 4 h, the protein was extracted and detected by Western blot analysis.

To investigate how CXCL14 regulates the NF-κB signaling pathway, we analyzed the above RNA sequencing data and screened out several genes, including SDC1, STAT1, STAT3, JAK1, and ERK1/2, which expression increased with CXCL14 overexpression in CECs. Then, the co-immunoprecipitation experiment was carried out, and we found that SDC1 binded to CXCL14 significantly among these proteins (Fig. 5B). Flag-tagged SDC1 and V5-tagged CXCL14 vectors were transfected into HCE-2 cells, and immunoprecipitation was performed with the indicated antibodies. Cell lysates and immunoprecipitated proteins were analyzed by immunoblot (IB) with the indicated antibodies.

### Specific experimental process

#### Coimmunoprecipitation assay

HCE-2 **(**50, B1) cells were seeded in a 10-cm dish and transiently transfected with 3 μg of pFLAG-plasmid and 3 μg of MYC-plasmid by Lipofectamine 2000 (Invitrogen, Carlsbad, CA, USA). After 24 h of post-transfection, cells were lysed by sonication in Immunoprecipitation (IP) buffer. IP assay was carried out by incubating the supernatants with an anti-FLAG antibody (Sigma, #SAB4301135) or an anti-MYC antibody (Cell Signaling Technology, #2276), followed by incubation with protein G agarose (Thermo-Fisher Scientific) at 4 °C overnight. Then, proteins bound to the beads were eluted by boiling in Sodium dodecyl sulfate (SDS) loading buffer and separated by SDS-PAGE for Western blot analysis to detect CXCL14 and SDC1.

### Chromatin immunoprecipitation assay

Chromatin immunoprecipitation assays were performed by using a ChIP kit (Millipore) according to the manufacturer’s instructions. Briefly, HCECs were cross-linked with 1% formaldehyde for 10 min at room temperature. After sonication and centrifugation, the supernatant was collected for anti-Flag immunoprecipitation. Anti-RNA polymerase and anti-rabbit IgG were also used as positive and negative controls, respectively. Semi-quantitative real-time PCR was performed to detect DNA fragments in the *CXCL14* promoter region. The PCR primers were as follows:

- CXCL14-F: TCATCTAGAATGAGGCTCCTGGCGGCCGCGCT
- CXCL14-R: TCAACGCGTCTACTCTTCGTAGACCCTGCGTTTC

### Dual-luciferase reporter gene detection assay

Vectors overexpressing PAX6 (pcDNA-PAX6) and control vectors (pcDNA-vector), along with luciferase expression vectors carrying mutations in the BM_c region (pGL3.1-CXCL14-HRE.MUT(c)), the BM_e region (pGL3.1-CXCL14-HRE.MUT(e)), and both BM_c and BM_e regions (pGL3.1-CXCL14-HRE.MUT(c + e)) of the CXCL14 gene promoter, were co-transfected into HCE-2 cells. pGL3.1-CXCL14 was used as a positive control, and pGL3.1 EV as a negative control, to analyze the impact of PAX6 overexpression on the transcriptional activity of the CXCL14 promoter.

HCE-2 cells were routinely cultured and replated at 50% density; when cells reached 70% confluency, they were transfected with the necessary plasmids for the luciferase report, along with corresponding transfection reagents. 48 h post-transfection, cells were lysed according to the lysis buffer instructions provided in the kit, and supernatants were collected post-centrifugation. The appropriate substrate was diluted as per the manual and protected from light. The prepared working solution was added to the cell supernatants to be tested. A luminometer was used to measure and compare the data, analyzing the impact on the binding sites.

### Immunofluorescence staining

Cells grown on coverslips were fixed with 4% paraformaldehyde and permeabilized in 0.25% Triton X-100 for 10 min at room temperature. After incubation with blocking buffer for 1 h, the cells were incubated at 4 °C overnight with anti-NF-κB antibodies (CST, #8242). Coverslips with cells were washed 3 times and then incubated with secondary antibodies (goat anti-rabbit IgG or goat anti-mouse IgG antibodies conjugated with Alexa488) for 1 h at room temperature. Cells were then washed and stained with 0.1 μg/mL DAPI (Sigma-Aldrich) and mounted onto glass slides with ProlongGold Antifade Reagent (Thermo-Fisher).

### Western blot analysis

Whole-cell extracts were lysed in cold RIPA lysis buffer supplemented with a protease inhibitor cocktail, and the lysate was subjected to SDS-PAGE. Proteins were transferred to nitrocellulose membranes (Millipore, Billerica, MA, USA) and blotted with antibodies against CXCL14, p63, NK-κB (p65), NK-κB (p50), Ki67, SDC1, Flag, PAX6, and β-actin.

### Real-time polymerase chain reaction

The RAN of central corneal tissue, limbal tissue, and kidney tissue of rats were extracted with TRIzol (Invitrogen) according to the manufacturer’s instructions. Then, the mRNA was reverse transcribed to cDNA using an real-time PCR system (TaKaRa), and real-time PCR were performed with Hieff qPCR SYBR Green Master Mix (Yeasen Biotech) and LightCycler 96 (Roche, Basel, Switzerland). The primers were as follows, and β-actin was used as a loading control.

- CXCL14-F: TCATCTAGAATGAGGCTCCTGGCGGCCGCGCT
- CXCL14-R: TCAACGCGTCTACTCTTCGTAGACCCTGCGTTTC

### Wound healing assay

Approximately 5 × 10^5^ cells were seeded in a 6-well plate. Wounds were generated by a conventional pipette tip when the cells were confluent. Images were taken immediately or at 6 h, 12 h, and 24 h post-wounding. Migration distance was determined by using Image J software and the wound healing rate is calculated as follows, the width of the unhealed wound/the initial width of the wound × 100%

### Cell colony formation assay

Cells were seeded in 6‐well plates at a density of 800 cells/well. After approximately 1 week, the cells grew to visible colonies and were stained with crystal violet. The colonies were counted under the microscope.

### Cell cycle assay

Cells were seeded in 6-well plates and fixed with 70% ethanol at 4 °C overnight. After washing, cells were resuspended and concomitantly treated with RNaseA (Sigma-Aldrich) and stained with 50 μg/mL of propidium iodide for 30 min. Cell cycles were analyzed by using a Beckman Coulter EPICS flow cytometer (Krefeld, Germany).

## Results

### CCECs independently facilitate corneal repair

**Fig. 1:**
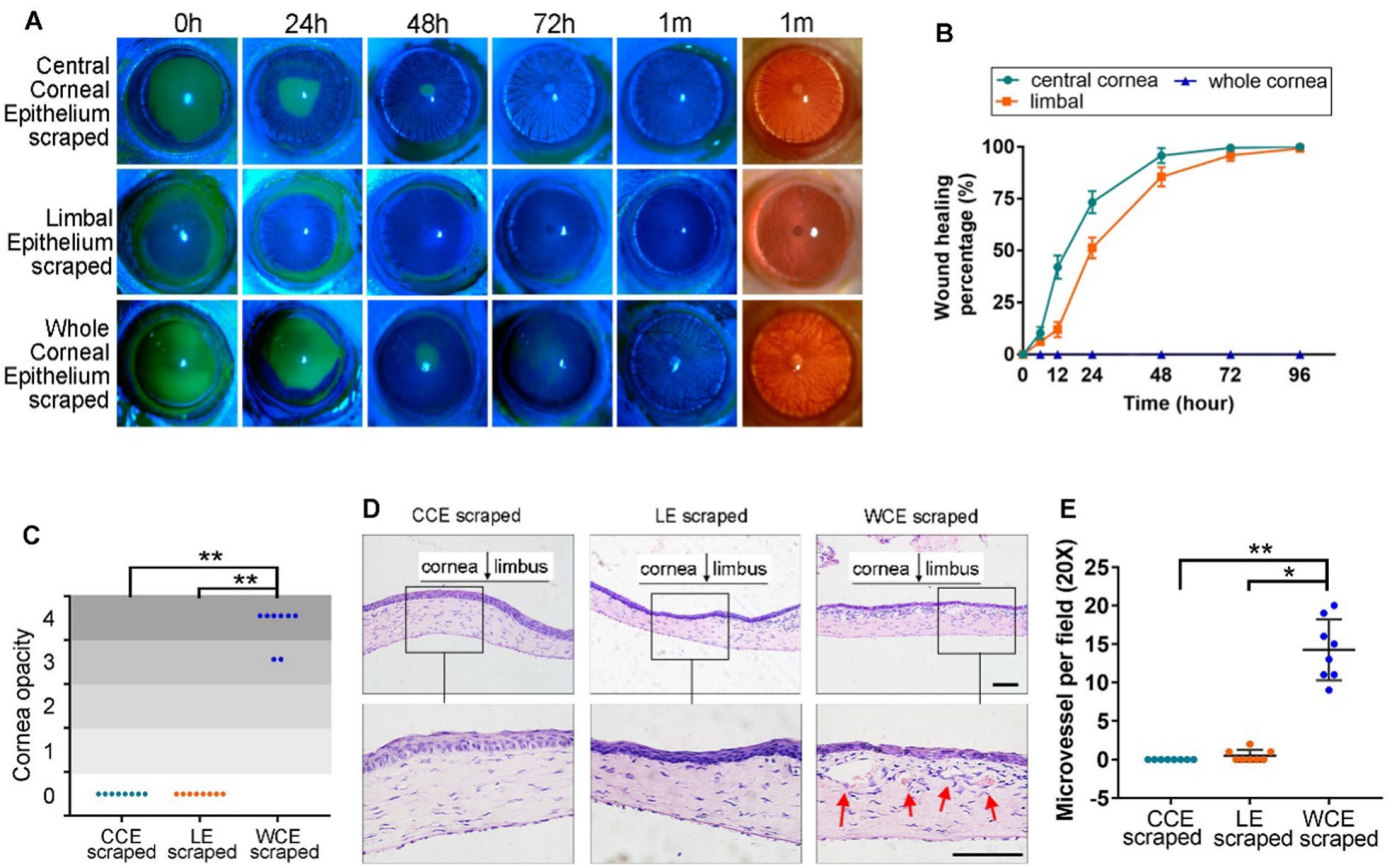
Wound healing of corneal epithelium in different model rats. **A** Representative image of eyes at different time points after corneal curettage injury in the 3 groups of rats. **B** The healing rate curve of corneal epithelium in the 3 groups of rats. **C** Grading of corneal turbidity in the 3 groups of rat eyes at 6 months postoperatively. **D** Hematoxylin–eosin staining of corneal tissue in the 3 groups of rat eyes at 6 m after operation. Red arrows indicate neovascularization. **E** The statistical results of microvessels in corneal tissue sections from the 3 groups of rat eyes at 6 m after operation. Statistical method: Mann–Whitney test. The data shown are presented as the mean ± SD. *P < 0.05; **P < 0.01. Scale bars, 100 μm

The results demonstrate the important role of CCECs in the repair of corneal injuries. The researchers established three corneal injury models and compared the repair capacity of CCECs to LECs and whole corneal epithelium (WCE) after mechanical curettage. We found that both CCECs and LECs were able to completely repair the corneal epithelium within 96 h after injury, as seen in Fig. 1A, B, while the WCE group required conjunctival tissue to cover the cornea for repair, resulting in corneal opacity and neovascularization, as seen in Fig. 1C–E. This indicates that CCECs have the ability to independently repair the cornea after injury.

### *CXCL14* is crucial for corneal repair, with high expression in CCE

**Fig. 2:**
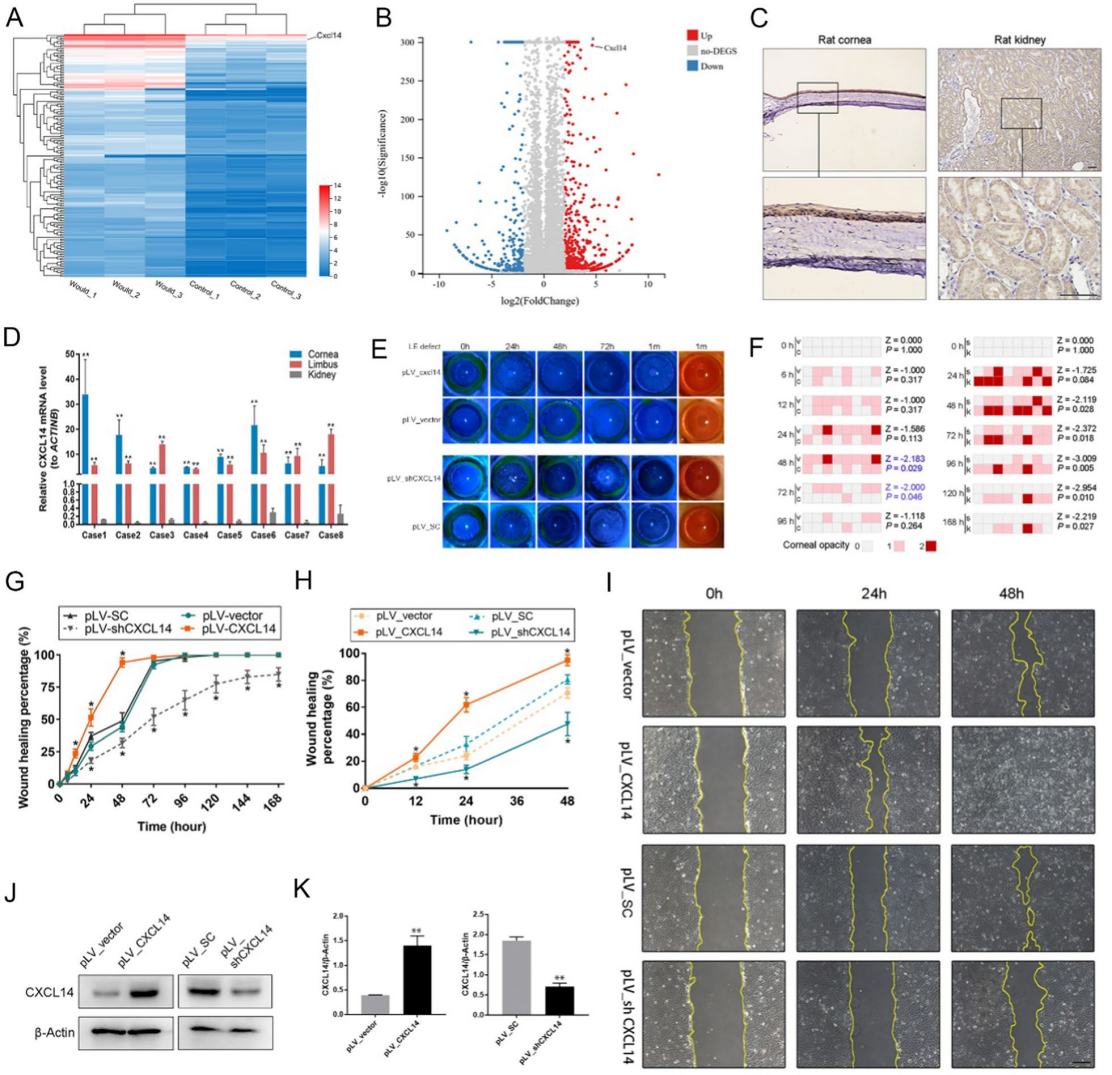
*CXCL14* is highly expressed in the cornea and promotes corneal repair after injury. **A** Upregulated gene cluster diagram: CXCL14 exhibits high baseline expression in CCE of normal rats. During the repair process of LE injury, the expression of CXCL14 in CCE specifically increases further. **B** Differential gene scatter plot from whole-genome sequencing: the expression of CXCL14 in the LE injury group is 25.63 times higher than its regular expression in the normal control group. **C** The immunohistochemistry staining of CXCL14 in the corneal tissues and kidney tissues of normal rats. **D** mRNA expression level of *CXCL14* in the central corneal tissue, limbal tissue, and kidney tissue of normal rats. **E** Representative photos of rats eyes, infected with different lentivirus after LE curettage. **F** The statistical results of corneal opacity from 4 groups of rat eyes after LE curettage [pLV_vector (V), pLV_CXCL14 (C), pLV_SC (S), pLV_shCXCL14 (K)]. **G** The healing rate curve of corneal epithelium in the above 4 groups of rat eyes. **H** Inter-group HCE-2 scratch assay healing curve line chart. **I** Representative images of wound healing assay in inter-group HCE-2. **J, K** Stable over/downregulated the expression of *CXCL14* was tested by Western blot analysis in HCE-2 cells infected with different lentivirus (**J**) and gray level histograms (**K**). Statistical method: Mann–Whitney test. Data are shown as the mean ± SD. *P < 0.05, **P < 0.01. Scale bars, 100 μm

To investigate which genes enhance the repair process of CCE in corneal injury, we established LE injury model in the left eye of rats, using the right eye as a normal control. CCE samples from both eyes were subjected to whole-genome sequencing analysis 24 h post-injury. The data revealed that CXCL14 is highly expressed in the CCE under normal conditions and shows a significant upregulation during the healing process, as seen in Fig. 2A, B. Meanwhile, the immunohistochemical and the real-time PCR results showed that *CXCL14* was highly expressed in the normal cornea tissues, especially in CCE tissues, and its expression was much higher than that in the kidney, which was used as a positive control (at present, it has been confirmed that *CXCL14* is highly expressed in the kidney [35]), as seen in Fig. 2C, D. Therefore, we speculated that *CXCL14* may play an important role in the repair of corneal epithelium.

The findings from the rat LE wound healing study indicate that overexpression of *CXCL14* enhances corneal wound healing and reduces corneal opacity during the recovery phase (within 48 h) compared to the control group. Conversely, suppression of *CXCL14* expression impeded corneal wound healing and increased corneal opacity, as seen in Fig. 2E–G (statistical analysis using Mann–Whitney test, *P* < 0.05). The HCECs wound healing results showed that the healing rate of the *CXCL14* over-expression group was significantly higher than that of the control group at 24 h and 48 h, while the healing rate of the *CXCL14* down-expression group was significantly lower than that of the control group, as seen in Fig. 2H–K (statistical analysis using Mann–Whitney test, *P* < 0.05).

These findings indicate that *CXCL14*, with highly expressed in CCECs, can effectively enhance the repair of corneal injury.

### *CXCL14* is crucial for promoting CCE proliferation

**Fig. 3:**
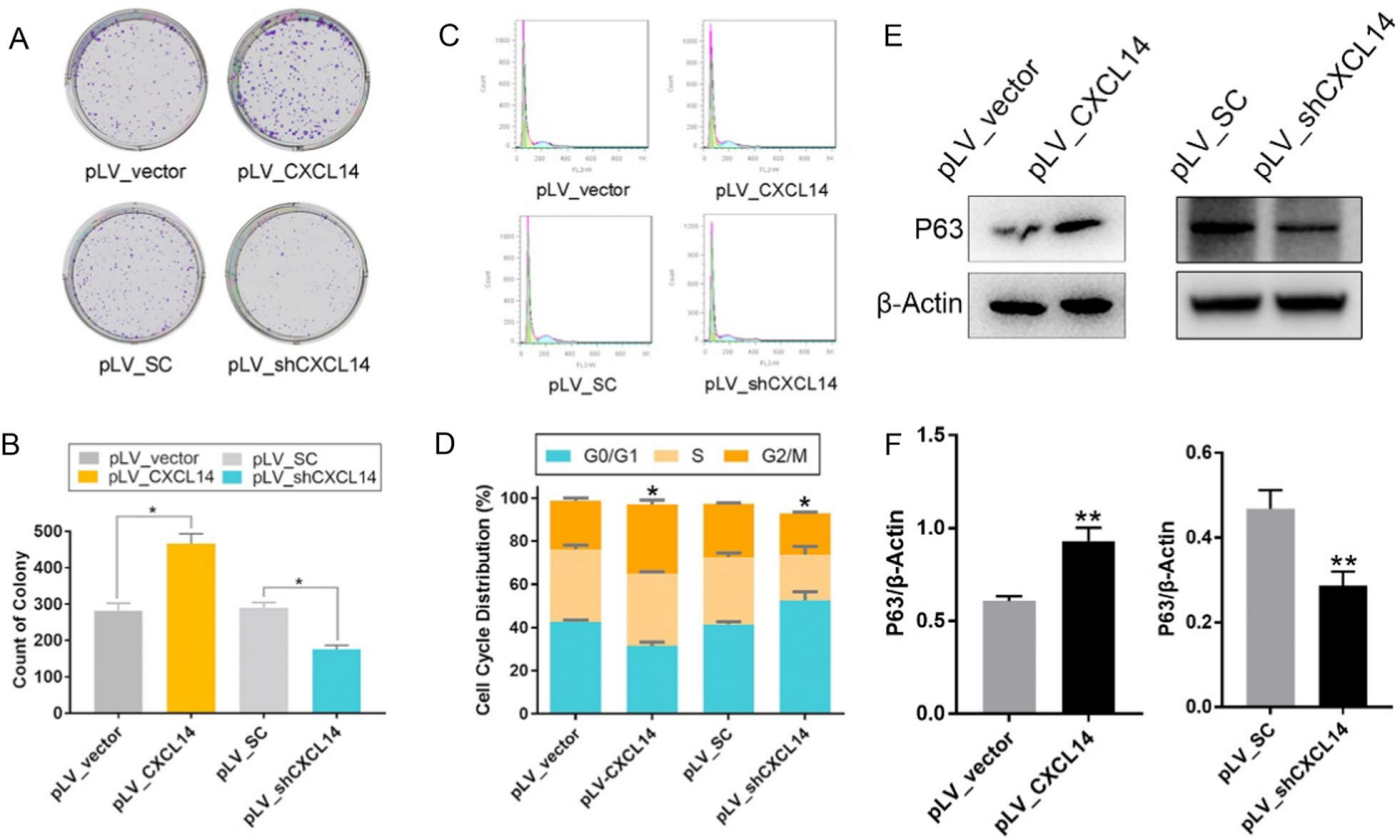
CXCL14 promotes CECs proliferation. **A, B** Representative images (**A**) and statistical results of soft agar (**B**) assay in HCE- 2 with upregulated or downregulated expression of CXCL14. **C, D** Representative cell cycle curves (**C**) and statistical results (**D**) of HCE- 2 with CXCL14over/downregulation. **E, F** Expression of p63 was detected in pLV_CXCL14 or pLV_shCXCL14 and control groups in HCE-2 by Western blot (**E**) and gray level histogram (**F**). Statistical method: Mann–Whitney test. Data are shown as the mean ± SD. *P < 0.05, **P < 0.01

The results revealed a significantly higher number of colonies in the *CXCL14*-upregulated group compared to the control, while the *CXCL14*-downregulated group exhibited notably fewer colonies. This suggests that *CXCL14* enhances CCECs proliferation, as seen in Fig. 3A, B.

The ratio of HCECs in the G0/G1 phase was decreased while that of HCECs in the G2/M phase was increased after overexpression of *CXCL14*, as seen in Fig. 3C, D. These results suggested that *CXCL14* can shorten the DNA synthesis prophase of CECs mitosis, promote DNA synthesis and replication, and promote CECs proliferation. Meanwhile, Western blot results for p63 (stemness marker) in CXCL14 over/down-pression and control groups in HCECs showed that *CXCL14* enhanced the stemness potential of CECs, as seen in Fig. 3E, F.

### *CXCL14* promotes CCE repair via activating the* NF-κB* pathway

**Fig. 4:**
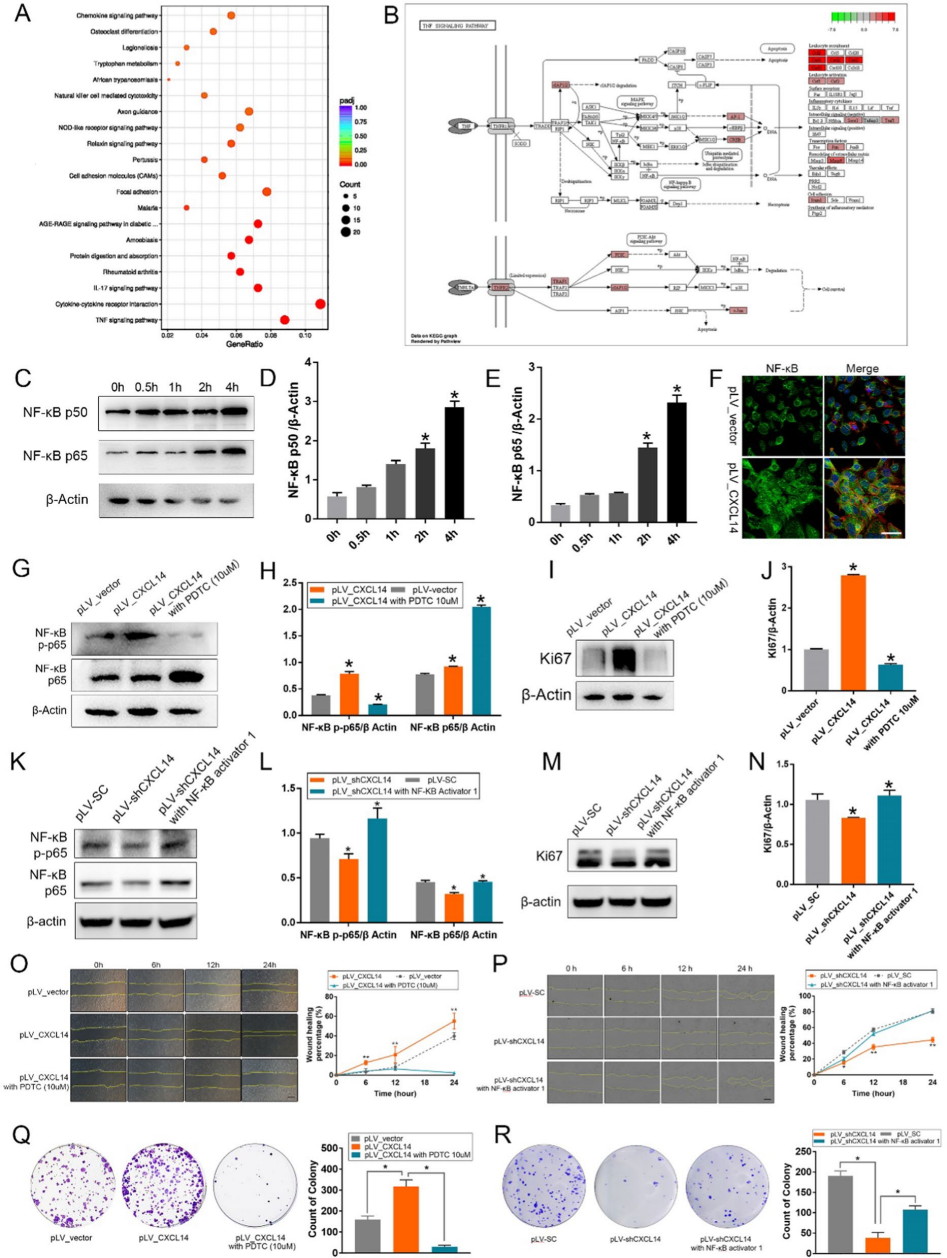
CXCL14 promotes the proliferation of CECs by activating the NF-κB signaling pathway. **A, B** RNA seq data of HCE-2 (pLV-CXCL14, pLV_vector): with the increased expression of *CXCL14*, the NF-κB signaling pathway was activated significantly. **C–E** The expression levels of NF-κB p50 and p60 were detected by Western blot analysis (**C**) and gray level histogram (**D, E**) after CXCL14 (20 ng/mL) stimulation in HCE-2. **F** The immunofluorescence staining results of NF-κB in HCECs with or without CXCL14 overexpression. **G, H** Western blot analysis (**G**) and gray level histogram (**H**) of NF-κB p65 and NF-κB p-p65 in pLV_CXCL14 HCECs with or without PDTC and control groups. **I, J** Western blot analysis (**I**) and gray level histogram (**J**) of Ki-67 in pLV_CXCL14 HCECs with or without PDTC and control groups. **K, L** Western blot analysis (**K**) and gray level histogram (**L**) of NF-κB p65 and NF-κB p-p65 in pLV_shCXCL14 HCECs with or without NF-κB activator and control groups. **M, N** Western blot analysis (**M**) and gray level histogram (**N**) of Ki-67 in pLV_shCXCL14 HCECs with or without NF-κB activator and control groups. **O, P** Representative images and line graphs of wound healing assay in pLV_CXCL14 HCECs with or without PDTC (**O**), in pLV_shCXCL14 with or without NF-kB activator and control groups (**P**). **Q, R** Representative images and statistical results of soft agar assay in pLV_CXCL14 HCECs with or without PDTC (**Q**), in pLV_shCXCL14 with or without NF-kB activator and control groups (**R**). Statistical method: Mann–Whitney test. Data are shown as the mean ± SD. *P < 0.05, **P < 0.01. Scale bars: 20 μm (**F**), 100 μm (**O, P**)

We conducted gene sequencing on HCE-2 cells with varying levels of *CXCL14* expression to identify downstream pathways involved in CCE repair mediated by *CXCL14*. Notably, there was a significant upsurge in the activity of the *NF-κB* signaling pathway linked to elevated *CXCL14* expression, as seen in Fig. 4A, B. P65 and p50 are the most common subunits of *NF-κB* [51]. Western blot analysis further confirmed that the expression of *NF-κB* p50 and particularly *NF-κB* p65 markedly increased following stimulation, as seen in Fig. 4C–E.

Through immunofluorescence analysis of HCE-2 cells, we observed that *CXCL14* overexpression markedly enhances the nuclear translocation of *NF-κB*, a crucial step for its activation, given that *NF-κB* is only active within the nucleus, as seen in Fig. 4F. Then protein samples were harvested from three HCE-2 cell groups: those with pLV_CXCL14-induced overexpression, those with both pLV_CXCL14 and the *NF-κB* inhibitor PDTC ((10 µM), and a control group. P65 is the most critical transcriptional activator within the *NF-κB* signaling pathway, and it requires phosphorylation to fulfill its functions of nuclear translocation, DNA binding, and transcriptional activation [51]. Consequently, western blot analysis was performed to assess NF-κB p65/p-p65 and Ki67 expression levels. Data indicate that overexpression of *CXCL14* led to an increase in the phosphorylation of *NF-κB* p65. Nevertheless, this enhancement in p65 phosphorylation was diminished upon the introduction of the *NF-κB* inhibitor PDTC, even in the context of CXCL14 overexpression. In parallel, levels of Ki67 escalated with the overexpression of *CXCL14* but were reduced by PDTC, as seen in Fig. 4G–J. The blockade experiment revealed that the downregulation of *CXCL14* inhibited the phosphorylation of *NF-κB* p65. However, when an *NF-κB* activator was introduced alongside the downregulation of *CXCL14*, there was a resurgence in the phosphorylation levels of p65. Meanwhile, the level of *Ki67* decreased following the downregulation of *CXCL14*, but saw a rebound when *CXCL14* was inhibited while *NF-κB* was activated, as seen in Fig. 4K–N. These findings indicate that *CXCL14* augments *NF-κB* p65 activity in CECs through boosting its nuclear import and phosphorylation, thereby promoting cell proliferation.

To investigate NF-κB’s role in CXCL14-mediated wound healing and proliferation in HCECs, we performed wound healing and soft agar assays on three groups: pLV_CXCL14, pLV_CXCL14 + PDTC, and control. *CXCL14* overexpression increased wound healing rate and HCECs proliferation, effects that were countered by *NF-κB* inhibition. Then, we performed wound healing and soft agar assays on three groups: pLV_shCXCL14, pLV_shCXCL14 + NF-κB activator, and control. *CXCL14* down-expression decreased wound healing rate and HCECs proliferation, this blocking effect disappeared after restoring *NF-κB* activity, as seen in Fig. 4O–R.

Overall, these data reinforce the notion that *CXCL14* stimulates CECs proliferation and repair through *NF-κB* signaling pathway.

### *SDC1* is identified as the receptor driving *CXCL14*-induced activation of the *NF-κB* pathway

**Fig. 5:**
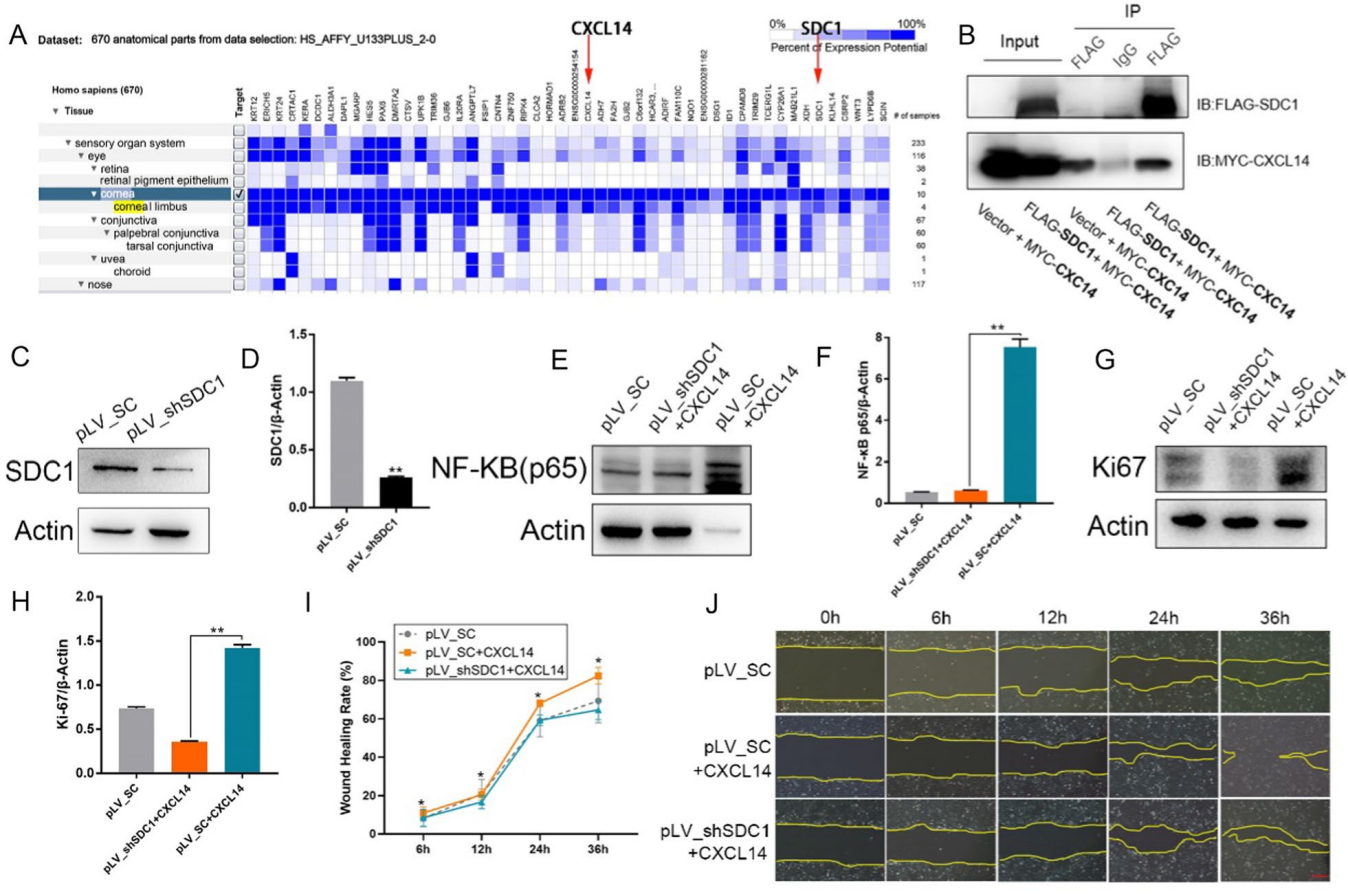
CXCL14 activates the NF-κB signaling pathway by binding to SDC1.** A** Human corneal gene sequencing database (GEO dataset GSE38190), by using Genevitator (www.genevestigator.com). **B** Co-IP assay confirmed the binding of CXCL14 to SDC1 in HCECs. **C, D** Western blot analysis (C) and gray level histogram (D) of SDC1 in HCE-2 with downregulated expression of *SDC1* and control. **E, F** Western blot analysis (**E**) and gray level histogram (**F**) of NK-κB p65 in HCE-2 treated with CXCL14 (20 ng/mL) with or without SDC1 downregulation and control. **G, H** Western blot analysis (**G**) and gray level histogram (**H**) of Ki67 in HCE-2 treated with CXCL14 (20 ng/mL) with or without SDC1 downregulation and control. **I, J** Representative images (**I**) and statistical results (**J**) of wound healing assay in HCE-2 treated with CXCL14 (20 ng/mL) with or without SDC1 downregulation and control. Statistical method: Mann–Whitney test. Data are shown as the mean ± SD. *P < 0.05, **P < 0.01. Scale bars, 100 μm

The analysis of the human corneal gene sequencing database (GEO dataset GSE38190) showed that both *CXCL14* and *SDC1* were highly expressed in corneal tissues, ranking among the top 50, as seen in Fig. 5A. Co-immunoprecipitation experiments in HCE-2 cells confirmed the interaction between *SDC1* and *CXCL14*, with MYC-tagged *CXCL14* co-precipitating with Flag-tagged *SDC1*, as seen in Fig. 5B. Additionally, *SDC1*’s activation is linked to *NF-κB* signaling, highlighting its significance in corneal function [43].

To determine if *CXCL14* activates *NF-κB* by binding to *SDC1*, HCE-2 cells with *SDC1* knockdown were created via lentiviral infection, as seen in Fig. 5C, D. Following treatment with recombinant *CXCL14* (20 ng/mL), western blot analyses revealed that *SDC1* suppression reduced *CXCL14*’s effect on both *NF-κB* and proliferation marker Ki67, as seen in Fig. 5E–H. Additionally, in vitro wound healing assays showed that *SDC1* knockdown significantly impaired *CXCL14*’s promotion of cellular repair, as seen in Fig. 5I, J.

These findings indicate that *CXCL14* modulates *NF-κB* signaling through *SDC1* binding, enhancing CECs proliferation and corneal repair post-injury.

### *PAX6 *directly regulates the expression of *CXCL14* in CECs

**Fig. 6:**
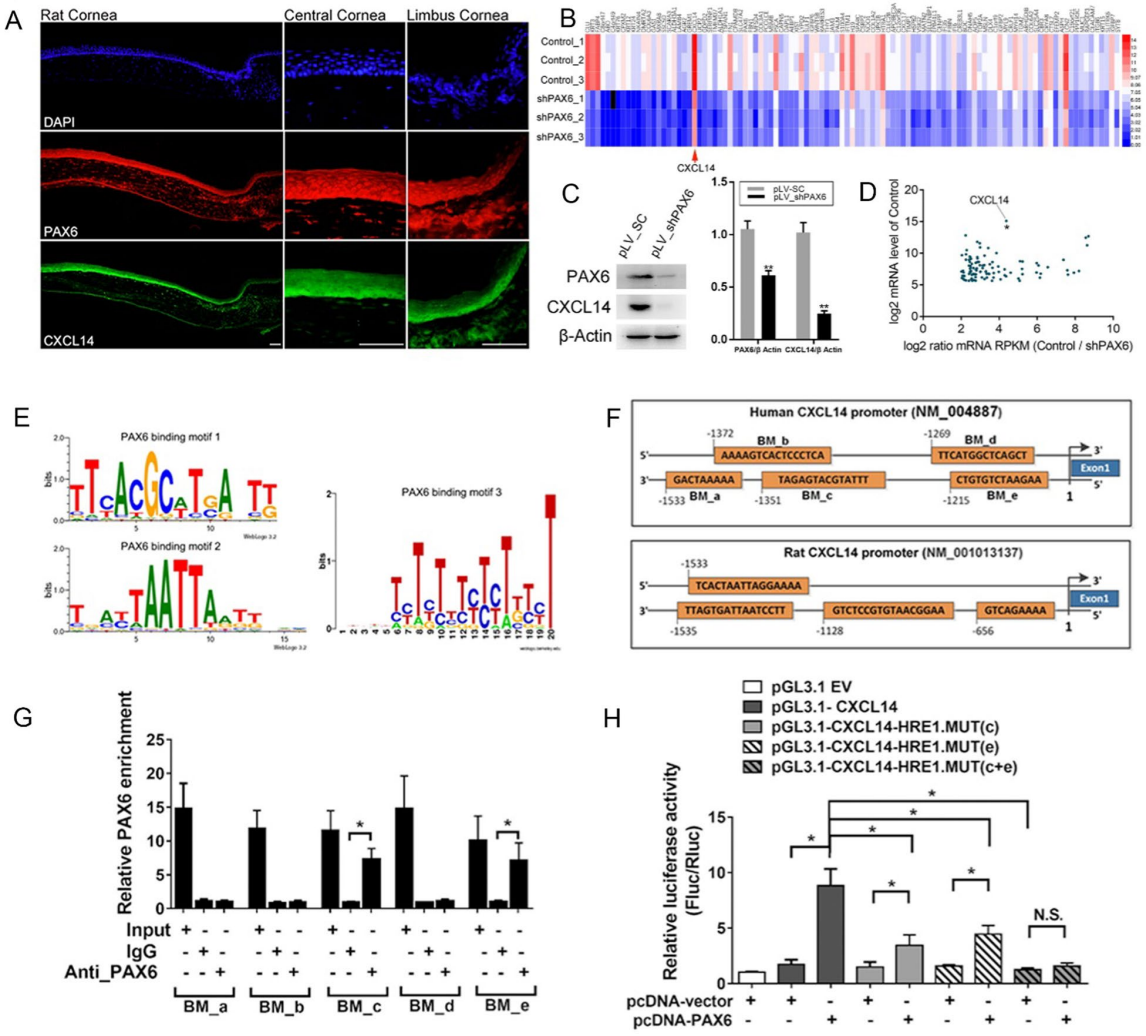
PAX6 directly regulates the expression of *CXCL14* in CECs. **A** The results of immunofluorescence staining shown the expression and localization of PAX6 and CXCL14 in normal rat corneas. **B** The heat map of RNA-seq results for the differential expression genes in HCECs with PAX6 silencing as compared with control cells. **C** Western blot analysis and gray level histogram of PAX6 and CXCL14 in HCECs with PAX6 downregulation and control. **D** The scatter plot of RNA-seq results for the differential expression genes in HCECs with PAX6 silencing as compared with control cells. **E** Schematic diagram of PAX6 binding motifs. **F** Potential PAX6 binding sites in the promoter region of the *CXCL14* gene in humans and rats (5 potential PAX6 binding sites in the promoter region of the human *CXCL14* gene were identified as BM_a-e to use as tag names to facilitate subsequent experiments). **G** Chromatin immunoprecipitation results confirmed that PAX6 strongly bind to 2 sites (BM_c, BM_e) in the promoter region of *CXCL14* in HCECs. **H** Daul luciferase assay results confirmed that PAX6 directly regulated CXCL14 expression by binding to BM_c and BM_e sites of *CXCL14* promoter. Statistical method: Mann–Whitney test. Data are shown as the mean ± SD. *P < 0.05, **P < 0.01. Scale bars, 100 μm

*PAX6* is a crucial regulatory gene for eye. It exerts influential control over numerous downstream target genes and signaling pathways. Following corneal injury, *PAX6* expression substantially increases to enhance repair and sustain CECs proliferation [21]. Immunofluorescence staining revealed that *PAX6* and *CXCL14* proteins were abundantly present in the WCE of healthy rats, with *CXCL14*’s distribution in the cornea mirroring that of *PAX6*, as seen in Fig. 6A.

To determine if *PAX6* modulates *CXCL14,* we developed a PAX6-knockdown HCE-2 cell line via lentiviral infection and confirmed via Western blot that *CXCL14* expression markedly decreased with *PAX6* suppression, as seen in Fig. 6C. RNA sequencing of these *PAX6*-downregulated cells revealed significant suppression of *CXCL14*, as seen in Fig. 6B, D, indicating *PAX6*’s regulatory role on *CXCL14* expression.

To elucidate *PAX6*’s regulatory mechanism on *CXCL14*, we examined their base sequences, identifying potential *PAX6* binding sites in the *CXCL14* gene promoters of humans and rats (Fig. 6E), suggesting a direct transcriptional regulation by *PAX6*. Using molecular bioinformatic analysis, we identified 5 potential *PAX6* binding sites between − 1533 bp and − 1215 bp upstream of the human *CXCL14* gene and named them as BM_a to BM_e (Fig. 6F). CHIP immunoprecipitation confirmed *PAX6*’s binding to BM_c and BM_e (Fig. 6G). In dual luciferase assays featuring overexpressed *PAX6* vectors and variously mutated *CXCL14* gene promoters, the following was observed: *PAX6* overexpression enhanced *CXCL14* promoter activity; mutations in BM_c or BM_e significantly dampened this activity; concurrent mutations in both BM_c and BM_e regions further suppressed transcription. These findings suggest that PAX6 specifically targets BM_c and BM_e within the *CXCL14* promoter to upregulate transcription, as seen in Fig. 6H.

### *CXCL14* is essential for corneal repair processes driven by *PAX6*

**Fig. 7:**
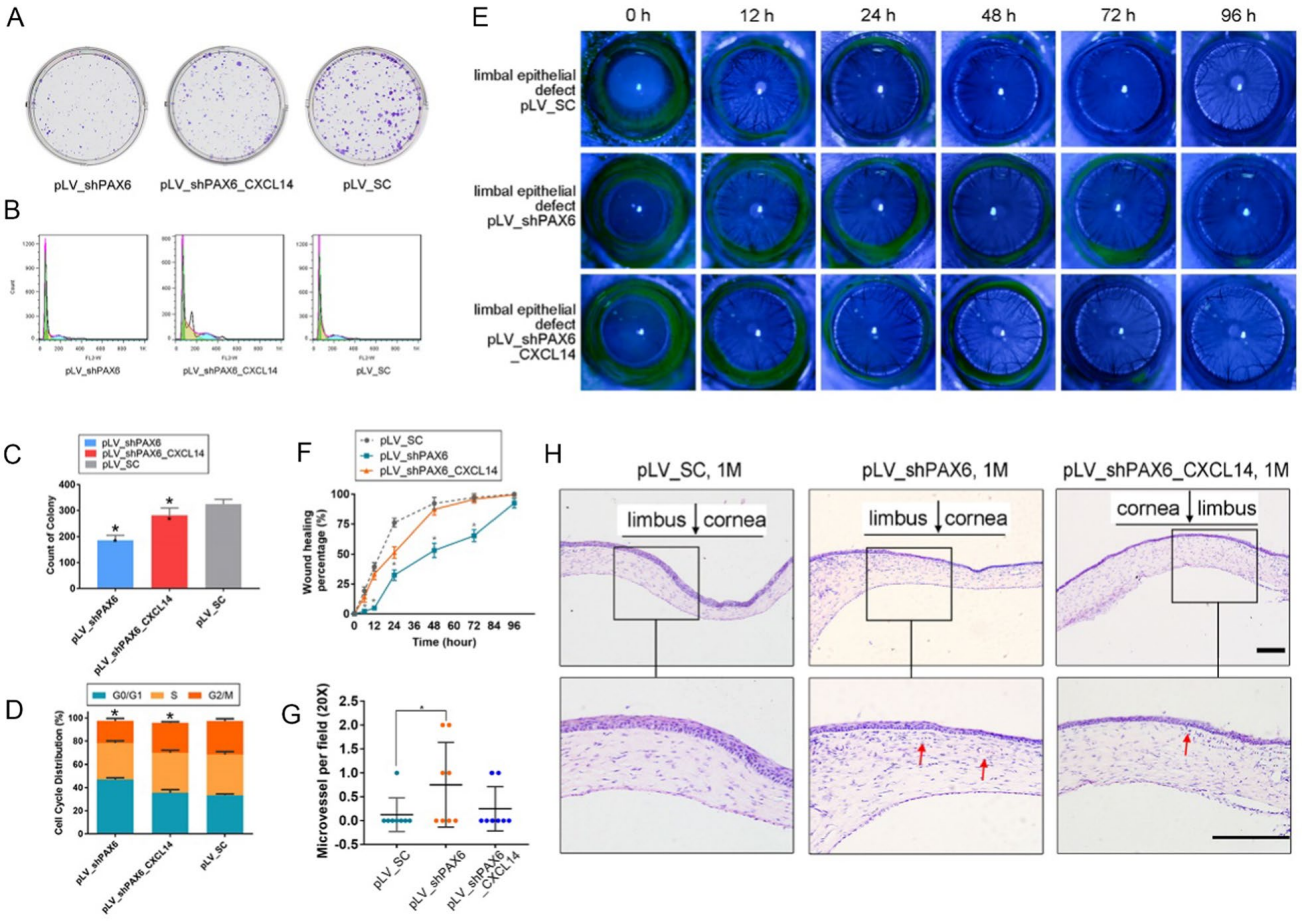
PAX6 promotes CEC repair of corneal injury by regulating CXCL14. **A, C** Representative images (**A**) and statistical results (**C**) of soft agar assay in HCE-2 (pLV_shPAX6, pLV_shPAX6 + CXCL14 (20 ng/mL), control). **B, D** Representative images (**B**) and statistical results (**D**) of cell cycle assay in HCE-2 (pLV_shPAX6, pLV_shPAX6 + CXCL14 (20 ng/mL), control). **E–H** Representative photos (**E**) and the healing rate curves (**F**) of rat eyes (pLV_shPAX6, pLV_shPAX6 + CXCL14, control), at 24 h, 48 h, 72 h, and 1 m after LE curettage, and hematoxylin–eosin staining (**H**) and the statistical results (**G**) of neovascularization in corneal tissue sections at 1 m after operation. Red arrows indicate neovascularization. Statistical method: Mann–Whitney test. Data are shown as the mean ± SD. *P < 0.05. Scale bars, 100 μm

To ascertain if *PAX6* facilitates corneal repair by directly modulating *CXCL14*, soft agar assays were conducted on *PAX6*-depleted HCE-2 cells with or without added recombinant human *CXCL14* (20 ng/mL). The findings revealed diminished proliferation in *PAX6*-suppressed HCECs, which recombinant *CXCL14* substantially recovered. Additionally, cell cycle analysis indicated that *PAX6*-downregulated HCECs had a reduced S phase population, yet recombinant *CXCL14* significantly bolstered their proliferation, as seen in Fig. 7A–D.

We created a rat LE injury model with reduced *PAX6* levels through lentivirus-mediated knockdown, followed by treatment with recombinant *CXCL14*. Observations with a slit lamp indicated that *PAX6* downregulation slowed corneal healing at multiple time points post-injury and prolonged edema and haze compared to the control group. *CXCL14* application, however, mitigated these effects, as seen in Fig. 7E, F. 1 m after operation, histological analysis revealed new blood vessel formation in the corneal tissue of four rats with *PAX6* knockdown, while only 2 rats in the pLV-shPAX6-CXCL14 group, as seen in Fig. 7G, H.

These results indicate that *PAX6* facilitates corneal repair by enhancing CECs proliferation through *CXCL14* regulation.

### Recombinant CXCL14 significantly promotes corneal repair

**Fig. 8:**
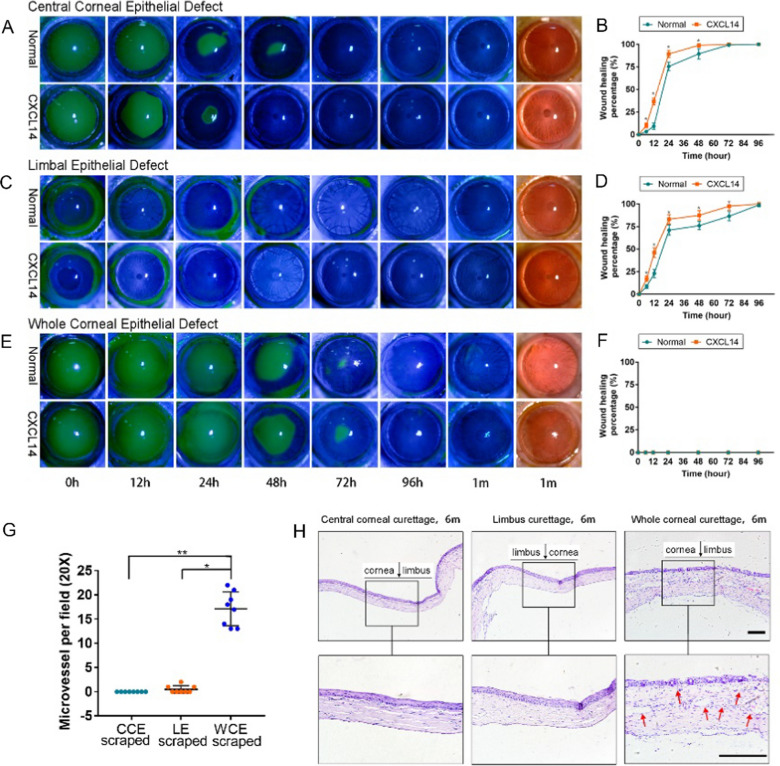
Recombinant CXCL14 promote corneal repair after injury. **A–F** Representative photos and the healing rate curve of rat eyes treated with recombinant human CXCL14 (20 ng/mL, Qid) or blank control: after CCE curettage (**A, B**), after LE curettage (**C, D**), after WCE curettage (**E, F**). **G, H** The statistical results of neovascularization of corneas treated (**G**) and HE staining images (**H**) with recombinant CXCL14 for 2 w in CCE, LE, and WCE curettage rats at 6 m after operation. The red arrows show a large amount of new blood vessels in the stromal and epithelial layers. Statistical method: Mann–Whitney test. Data are shown as the mean ± SD. *P < 0.05; ** *P* < 0.01. Scale bars, 100 μm

To verify if recombinant CXCL14 aids in corneal repair, we formed 3 groups of rat models with different corneal injuries (CCE scraped, LE scraped, and WCE scraped) and treated them with recombinant CXCL14 (20 μg/mL) or a blank control. Slit lamp images revealed that CXCL14 significantly improved repair in CCE and LE injuries, reducing corneal edema and haze more effectively than the control group, as seen in Fig. 8A–F. After healing, the corneas remained clear and stable for an extended period. However, in the WCE scraped group, despite treatment with CXCL14, wounds did not heal, resulting in corneal conjunctiva, opacity, and neovascularization, as seen in Fig. 8G, H.

These results suggested that the recombinant CXCL14 promotes the repair and healing of corneal injury and maintains the stable state of the cornea after wound healing.

## Discussion

The cornea, as a crucial component of the ocular surface, plays a vital role in maintaining the structure and optical properties of the eye. The corneal epithelium, as the first physical barrier of the cornea, is essential for corneal repair and tissue homeostasis. While LSCs have been extensively studied for their role in corneal repair and maintenance, there is increasing evidence that CECs also contribute to the healing process. Our study focused on *PAX6* and its downstream target *CXCL14*, elucidating their roles in corneal repair.

Corneal epithelium homeostasis entails continual self-renewal [22]. While most studies concentrate on LSCs for their critical role in sustaining corneal function and injury repair due to their undifferentiated nature, CCECs also express the stemness marker p63, albeit less than LSCs [23–28]. Despite LSCs taking 4–5 months to proliferate and migrate centrally post-injury, immediate and notable repair occurs, suggesting CCECs’ significant involvement in both corneal repair and homeostatic maintenance [29]. Several independent research teams have discovered that corneas in various animals, including humans, rabbits, mice, and pigs, can fully heal and retain transparency even without LSCs [15–19]. Majo et al., in a *Nature* publication, suggest this may be due to the presence of other stem-like cells in the corneal epithelium’s basal layer, which also contribute to corneal homeostasis and repair [19]. Although these studies highlight the crucial function of CCECs in independently repairing the cornea after injury, the precise mechanisms behind this remain unclear.

To investigate CCE’s unique role in corneal repair, we created three corneal injury model groups: CCE, LE, and WCE, each scraped off respectively. We observed that in the absence of LE, CCE alone could fully repair the injury, maintaining corneal transparency and stability up to six months post-operation. During this period, *CXCL14*, initially highly expressed in the corneal epithelium, showed increased specificity in expression during corneal repair post-injury. Currently, *CXCL14*’s role in the cornea is unreported. Its major amino acid sequence is highly conserved across vertebrates [29], and it is known to enhance proliferation and migration in various cell types in other tissues [30–37]. Therefore, we conducted a targeted study on the function of *CXCL14* in corneal repair by CCE, and confirmed that *CXCL14* promotes the proliferation of HCECs by promoting DNA replication and stemness. We also found that *CXCL14* promotes the role of CCE in repairing the cornea through the wound healing assay in vitro and the corneal injury model with lentivirus infection in vivo.

Gene sequencing analysis indicated a significant activation of the *NF-κB* pathway, marked by increased *CXCL14* expression. *CXCL14* stimulation enhanced nuclear expression of *NF-κB* p50 and p65. Inhibiting *NF-κB* with PDTC counteracted *CXCL14’*s effects on *NF-κB* p65 phosphorylation and Ki67 expression, a proliferation marker. Wound healing and soft agar assays established the dependency of *CXCL14*-driven repair and proliferation on *NF-κB* signaling. However, *CXCL14* is the only chemokine whose receptor has not yet been identified [38]. To explore the mechanism by which *CXCL14* promotes repair of corneal injury in CCECs, we screened potential receptors through bioinformatics analysis methods and found that *CXCL14* binds to *SDC1* through co-immunoprecipitation assay [44, 45]. We also confirmed that *CXCL14* could promote the proliferation and repair of HCECs by binding with *SDC1*. Subsequently, Western blot results showed that *CXCL14* can regulate the NF-κB signaling pathway by binding to *SDC1*.

*PAX6* stands as a cornerstone gene in ocular genetics, operating as a rare master regulatory protein that orchestrates eye morphogenesis and occupies a supreme position in the hierarchy of ocular genetics. Teaming up with genes like *BSAP/Pax5, Gata1, Gata2, MyoD, PU.1, Runx2, and Sox9, PAX6* functions as a paramount "molecular switch" within the extensive genetic network. It exerts influential control over numerous downstream target genes and signaling pathways, playing a pivotal role not only in the development and normal physiological functioning of the cornea but also in the healing processes following corneal injuries. When corneal injury occurs, *PAX6* expression is significantly upregulated to promote repair, and the *PAX6* has the ability to maintain the proliferation of CECs [21]. It controls cell type and differentiation by regulating various downstream genes. And it maintaines a stable high expression state in CECs and is related to the regulation of corneal repair. It has been reported that the downregulation of *PAX6* can cause changes in the biological characteristics of CECs, leading to delayed repair. However, to date, there is no report about the target genes regulated by *PAX6* affect corneal repair in CECs [39–41]. We found that the localization of *CXCL14* in the cornea was consistent with that of *PAX6*, and *CXCL14* expression decreased significantly with *PAX6* downregulation. Bioinformatics analysis results showed that there were potential binding sites of *PAX6* in the promoter region of *CXCL14*. And we confirmed that *PAX6* could directly bind to *CXCL14* promoter by chromatin immunoprecipitation assay and dual-luciferase analysis. We also verified that *PAX6* can promote the proliferation of CECs and corneal repair by regulating *CXCL14*.

Our study suggested that *PAX6* transcriptionally regulated *CXCL14* expression, and then *CXCL14* binded to *SDC1* to activate the NF-κB signal pathway, thereby promoting CEC proliferation and corneal repair. *CXCL14* is a secreted water-soluble protein. We tried to apply recombinant *CXCL14* eye drops to the rat corneal injury model. Through this study, we found that recombinant *CXCL14* eye drops can effectively promote the repair of corneal injury, and the corneal tissue structure and morphology did not change significantly after using them. Considering the high expression of *CXCL14* in the corneal epithelium, the application of *CXCL14* eye drops on the cornea is safe and non-toxic. Therefore, recombinant *CXCL14* may emerge as a novel therapeutic agent for the repair of corneal injuries. However, traditional eye drops face numerous limitations, such as the eye’s unique anatomical barriers that result in short residence times of drugs on the ocular surface, rapid release, and few drugs penetrating the ocular barriers. This leads to low bioavailability of the drugs, necessitating frequent administration, which in turn can reduce patient compliance. To address the bottlenecks in treating ocular diseases with eye drops, researchers have recently introduced nanotechnology into the preparation process of eye drops. These nanomedicines offer advantages such as enhanced solubility, improved bioavailability, extended circulation time, and targeted drug delivery [46, 47]. Additionally, the properties of the nanomaterial scaffolds match those of the natural tissue’s cellular matrix, allowing for optimal integration with the target tissue [48]. Regarding the application of recombinant *CXCL14* protein as a new type of drug for ocular use, we are considering combining it with nanomaterials for further research in the future.

## Conclusion

**Fig. 9:**
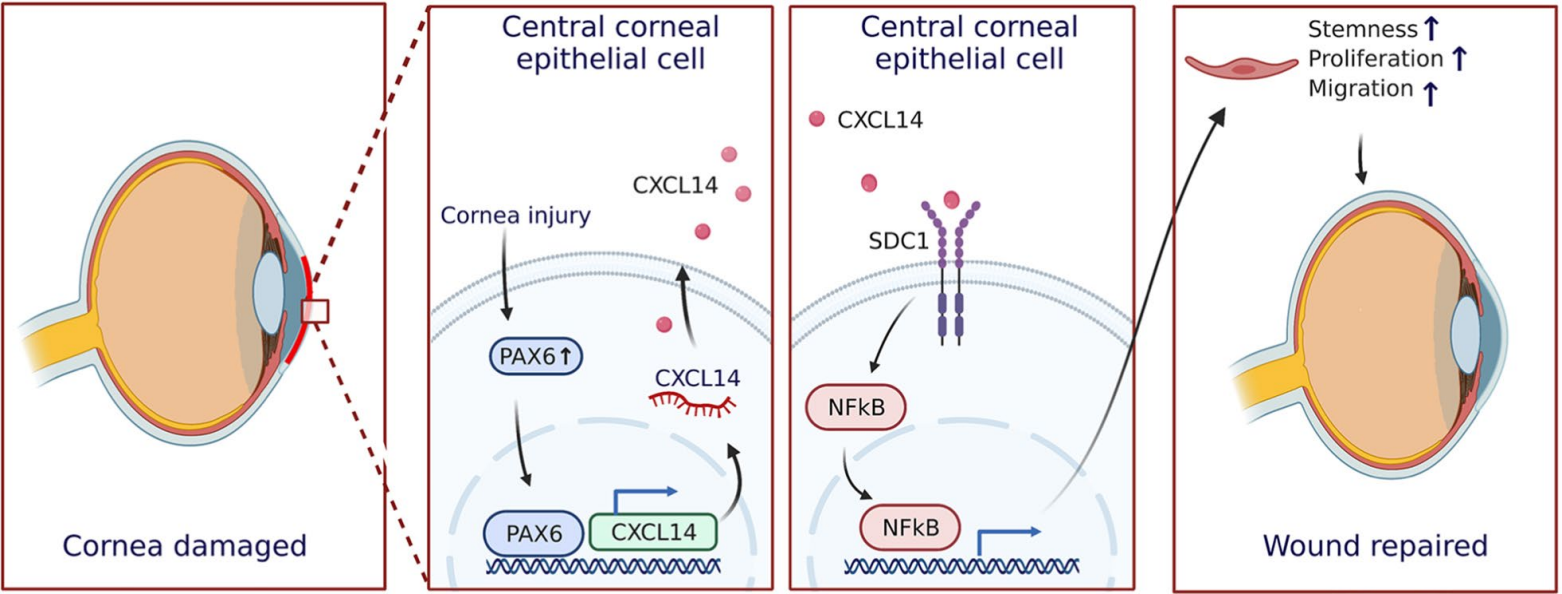
Schematic diagram of the mechanism for corneal injury repair mediated by the *PAX6/CXCL14* regulatory axis

Our results showed that CCECs were cells with stem potential and proliferation ability, which played important roles in corneal repair and in maintenance of corneal tissue homeostasis independently of LSCs. *PAX6* was first found to directly transcriptionally regulate *CXCL14*, and it was first confirmed that *CXCL14* can activate the NF-κB signal pathway through its receptor *SDC1* to promote CEC proliferation and corneal repair, which further proved that *CXCL14* has clinical application potential, as seen in Fig. 9.

However, our experiment is not without its limitations. The specific binding mode of *CXCL14* and *SDC1* and the specific regulation mode of *CXCL14* on NF-κB have not been clarified, and these studies will continue in the future. Moreover, there is a need for continued research into the application of *CXCL14* in conjunction with nanomaterials for use in eye drops.

In addition, although the exact location of human LSCs has been known for more than 20 years, it is still difficult to determine the exact location of LSCs in other animals because of differences in corneal epithelial tissue structures between humans and other mammals. Therefore, the rat model of LSC loss established by scraping the limbal epithelium is bound to be different from that of human LSC loss. However, some lineage tracing studies have shown that LSCs were also exist in mice, although their tissue structure is different from that of humans [42]. Therefore, it is of great significance and reference value to establish an LSC loss model in rats when the model cannot be established through human eyes.

### Supplementary Information

Supplementary Material 1.

## Data Availability

All data associated with this study are available from the corresponding author upon request.
