# Supplementary material for: PAX6/CXCL14 regulatory axis promotes the repair of corneal injury by enhancing corneal epithelial cell proliferation
Source: J Transl Med. 2024 May 15;22:458. doi: 10.1186/s12967-024-05270-z (PMC11094923; doi:10.1186/s12967-024-05270-z)

**Supplementary Figure 1. Establishment of rat corneal models with CXCL14 overexpression or downregulation by lentivirus infection. a** Immunohistochemistry staining of CXCL14 in rats cornea infected with pLV-CXCL14 or pLV-vector at 1d and 7d after infection. **b** Immunohistochemistry staining of CXCL14 in rats cornea infected with pLV-shCXCL14 or pLV-SC at 1d and 7d after infection. **c** HE staining of rats cornea tissues with or without lentivirus infection.


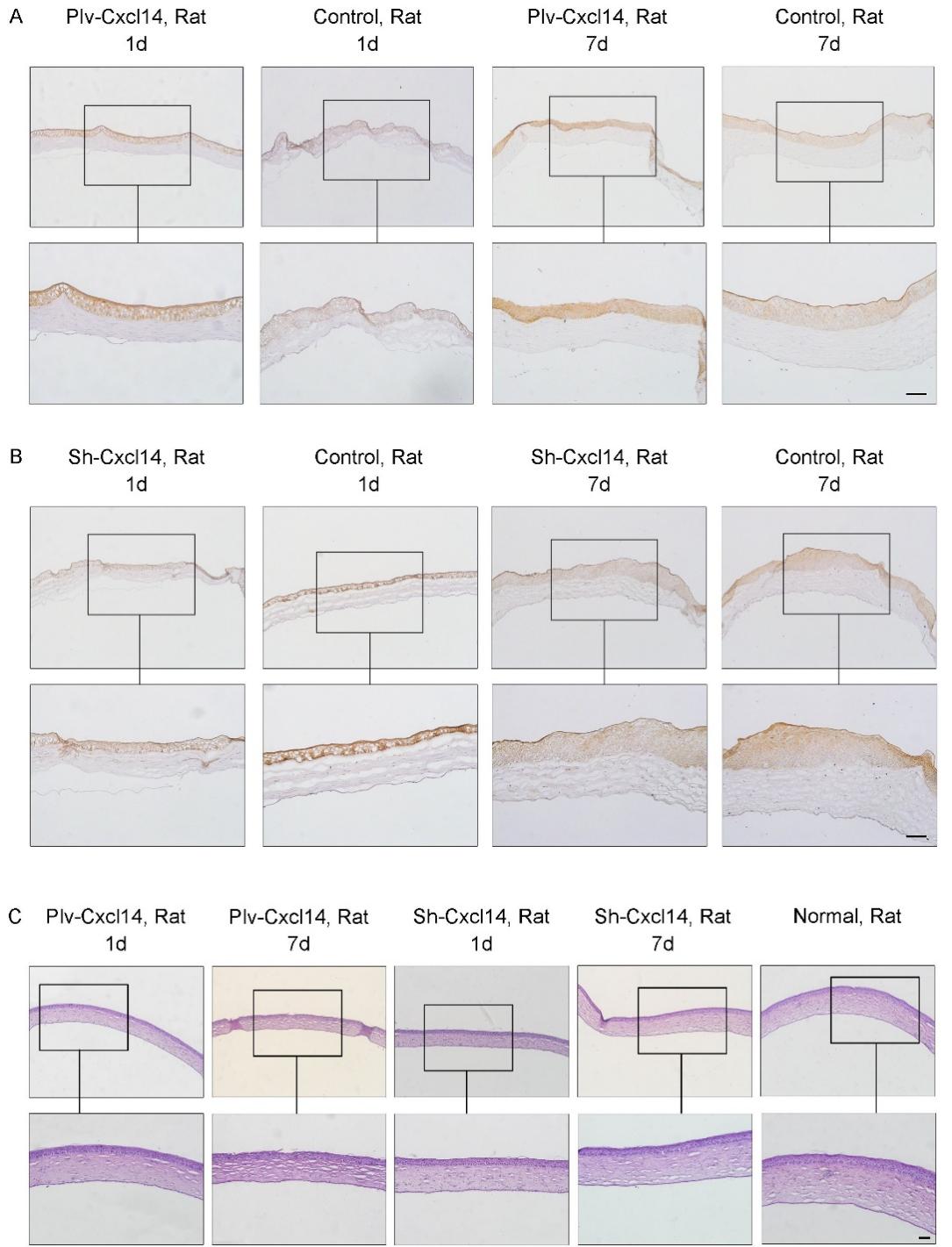

Supplement: Supplementary file 1 — Supplementary Material 1. [file 12967_2024_5270_MOESM1_ESM.docx]
